# Color Stability of Orthodontic Ceramic Brackets and Adhesives in Potentially Staining Beverages—In Vitro Study

**DOI:** 10.3390/dj10070115

**Published:** 2022-06-22

**Authors:** Luka Šimunović, Tadeja Blagec, Andrea Vrankić, Senka Meštrović

**Affiliations:** 1 School of Dental Medicine, University of Zagreb, Gundulićeva 5, 10000 Zagreb, Croatia; lsimunovic@sfzg.hr (L.Š.); avrankic@sfzg.hr (A.V.); 2 Department of Orthodontics, School of Dental Medicine, University of Zagreb, Gundulićeva 5, 10000 Zagreb, Croatia; tblagec@sfzg.hr

**Keywords:** ceramic brackets, adhesives, staining, color stability, spectrophotometry, esthetics

## Abstract

The aim of this study was to evaluate the color stability of esthetic ceramic brackets and adhesive samples after immersion in most commonly consumed beverages. A hundred ceramic brackets from five different manufacturers (Forestadent^®^, G&H^®^, GC, DynaFlex^®^, and American Orthodontics) and 120 samples of adhesives (3M™Transbond™ XT and American Orthodontics BracePaste^®^ color change adhesive and BracePaste^®^ adhesive) were immersed into four different solutions: coffee, Coca-Cola^®^, the vitamin drink Cedevita^®^, and artificial saliva (control group). The samples were kept in an incubator at 37 °C. Color readings were evaluated before (T0), at 24 h (T1), 72 h (T3), 7 days (T4), and 14 days (T5) after initial immersion using a spectrophotometer according to the L*a*b* color scale. All the examined brackets showed a statistically significant difference in discoloration (*p* = 0.001). 20/40™ Brackets (American Orthodontics) showed the best color stability, while the greatest color modification was recognized in QuicKlear^®^ III (Forestadent^®^) brackets. Regarding adhesives, the greatest staining was observed in the BracePaste^®^ color change adhesive and the least in the Transbond™ XT samples. In conclusion, color change occurs in all solutions, including control groups, and coffee has the greatest impact on color stability.

## 1. Introduction

To increase patients’ acceptance and to satisfy the esthetic demands of modern times, brackets should be invisible. There are a lot of different types of esthetic brackets; they can be either plastic or ceramic. Ceramic brackets have a better appearance and color stability [1].

Color stability can be defined as “the ability of a material to maintain its initial color for a certain period of time in response to a variety of factors in a particular environment and is an important physical feature for dental materials” [2,3]. 

The color modification of dental materials is a multifactorial process. The discoloration can be a result of multiple intrinsic and extrinsic factors. Consumption of pigmented food and drinks, using colored mouth rinses, smoking, poor oral hygiene and plaque are included in extrinsic discoloration factors [3,4,5,6]. 

Intrinsic discoloration depends on the degree of polymerization of resins and adhesives, water absorption, the composition of the material, and the size and content of filler particles [3,5,7]. Moreover, UV radiation, changes in temperature and humidity, and aging of the material are also potential threats to the color stability of esthetic brackets and composite resin [6,8,9,10]. 

Today’s high consumption of food and beverages full of natural and synthetic colors can result in the unsatisfactory esthetic performance of dental materials. Previous studies confirmed that esthetic brackets and adhesives are not color-stable in the long term and are susceptible to pigments commonly found in food and drinks [2,3,4,5,6,7,8,9,10].

Therefore, the aim of this study was to evaluate the color stability of esthetic ceramic brackets and adhesives for bracket bonding after immersion in the most commonly consumed beverages.

## 2. Materials and Methods

### 2.1. Brackets, Adhesives and Solutions

For this experimental study, 100 commonly used ceramic orthodontic brackets from 5 different manufacturers (Table 1) and 120 adhesive samples were immersed into four different solutions: coffee (Franck Jubilarna original, Franck d.d., Zagreb, Croatia), Coca-Cola (Coca-Cola HBC Hrvatska, Zagreb, Croatia), the vitamin drink Cedevita^®^ (Atlantic Grupa, Zagreb, Croatia), and synthetic saliva (Glandosane^®^ natural flavor, Stadapharm GmbH, Bad Vilbel, Germany), which was used as a control.

Three different adhesives were used: BracePaste^®^ adhesive and BracePaste^®^ color change adhesive (American orthodontics, Sheboygan, WI, USA) and Transbond™ XT light cure adhesive paste (3M™ Unitek, St. Paul, MN, USA). Adhesive specimens were made in molds of dimensions 4 × 2 mm at room temperature. The molds were pressed against glass slides on each side to remove the excess material and to provide a smooth surface of the specimen. The samples were cured with LED light (VALO™ Cordless, Ultradent Products, Inc., South Jordan, UT, USA, /395–400 nm/1000 mW/cm^2^) for 20 s on each side according to the manufacturer’s instructions. 

Five brackets from the same manufacturer and 10 samples of the same adhesive brand were immersed in each solution.

The content was kept in an incubator at 37 °C (Cultura, Ivoclar Vivadent, Schaan, Liechtenstein). Because of evaporation, the solutions were renewed after every 24 h of storage during the experimental period. 

### 2.2. Spectrophotometric Analysis

The color readings were estimated with a digital spectrophotometer (VITA Easyshade^®^ Advance 4.0, Vita Zahnfabrik, Bad Sackingen, Germany) positioned perpendicularly to the labial surface of the bracket and adhesive sample. Measurements were made on a mirrored surface, as this surface did not influence the color of the bracket on a black-andwhite surface [11,12,13]. The color readings were assessed according to the Commission Internationale de lEclairage (CIE) L*, a*, b* (LAB) color scale. L* value measures the amount of lightness, and a* and b* represent the four unique colors of human vision: red, green, blue, and yellow [14].

Before the reading, each bracket was taken out from the medium and washed out with a saline solution and properly dried on paper towels. All measurements were established under the same room lighting conditions. Before each measurement, the spectrophotometer was calibrated according to the manufacturer’s recommendations. The color of each bracket was detected three times without moving the position of the spectrophotometer. Averages for the values of L*, a*, and b* were evaluated.

Spectrophotometric readings were made at T0 (before initial immersion), T1 (24 h after initial immersion), T2 (72 h), T3 (7 days) and T4 (14 days after initial immersion). Total color change (Δ E*) was calculated by the following equation: ΔE* = [(ΔL*)2 + (Δa*)2 + (Δb*)2]1⁄2 [15]. Changes in color parameters (ΔL*), (Δa*), (Δb*) were calculated by subtraction of the final values from the baseline measurements (T0).

### 2.3. Statistical Analysis

Statistics were carried out using a Statistica 14.0.0.15 software package (TIBC Statistica Software Inc., Palo Alto, CA, USA).

Descriptive statistics, including mean, standard deviation, and minimum and maximum values were calculated for each group. A Kolmogorov–Smirnov test was applied to check whether distribution was normal within each group and a one-way analysis of variance (ANOVA) test was performed. A Kruskal–Wallis test was used to compare the differences in color changes among the groups. Significance for all statistical tests was at *p* ≤ 0.05.

## 3. Results

All the examined brackets showed a statistically significant difference in discoloration (*p* = 0.001). 20/40™ Brackets showed the best color stability, while the greatest color modification was recognized in QuicKlear^®^ III brackets.

**Figure 1:**
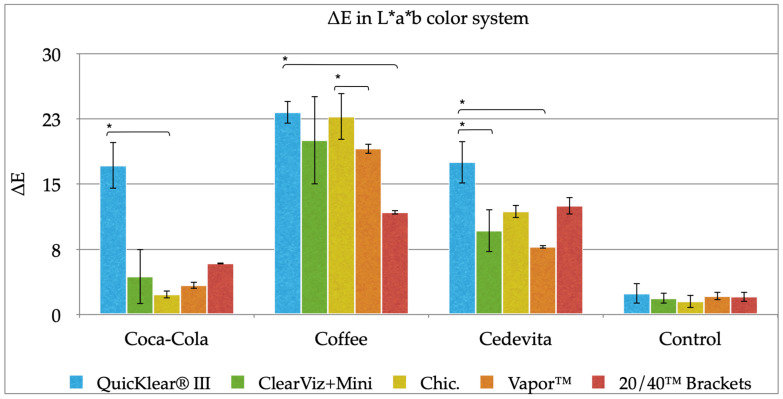
Color change values (ΔE*) of ceramic brackets after 14 days of immersion. * statistically significant difference *p* < 0.05.

**Figure 2:**
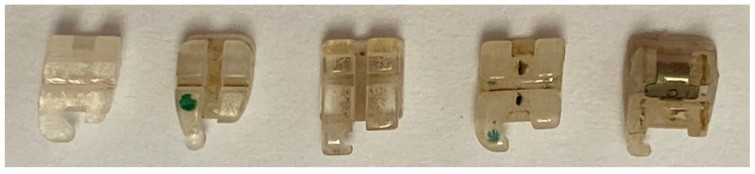
20/40™ Brackets, Vapor™, DynaFlex^®^, ClearViz + Mini and QuicKlear^®^ III^®^ brackets after 14 days of immersion in the coffee solution.

The highest color alteration was observed in the coffee solution for all bracket brands except for 20/40™ Brackets (Figure 1; Table 2). Color modifications after immersion in this solution are shown in Figure 2. Means and standard deviations of L* value, a* value and b* value of L*, a*, b* (LAB) color scale for ceramic brackets, according to the solution over time are presented in Supplementary Tables S1–S3.

QuicKlear^®^ III and Chic. brackets showed statistically significant higher discoloration in comparison to 20/40™ brackets after 14 days of immersion (*p* = 0.0043; *p* = 0.0094).

All brackets showed the lowest staining in Coca-Cola. There was also a significant difference in color modification between bracket brands. Chic. brackets had better color stability than QuicKlear^®^ III brackets (*p* = 0.0013).

In addition, QuicKlear^®^ III brackets immersed in the vitamin drink (Cedevita^®^) experienced greater color change in comparison with Vapor™ (*p* = 0.0005) and ClearViz + Mini brackets (*p* = 0.026).

Among all brackets and all solutions, only Vapor™ and Chic. brackets showed satisfying and clinically acceptable color stability after 14 days of immersion in Coca-Cola (ΔE* < 3.7).

All adhesive samples showed color alteration in all solutions over time. Transbond™ XT adhesives demonstrated the best color stability, while the greatest staining was observed in BracePaste^®^ color change adhesive.

**Figure 3:**
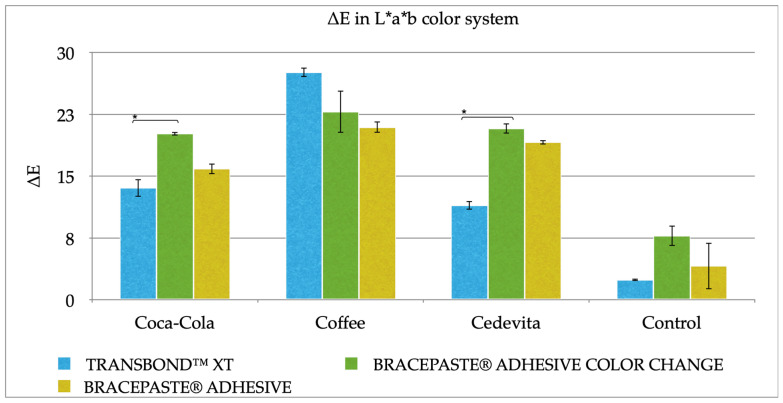
Color change values (ΔE*) of adhesive samples after 14 days of immersion. * statistically significant difference *p* < 0.05.

The greatest color change for all adhesive samples was found in the coffee solution (Table 3; Figure 3). Statistically significant color change was noticed in Coca-Cola and the vitamin drink (Cedevita^®^). Transbond™ XT adhesive samples showed greater color stability than BracePaste^®^ color change adhesive (*p* = 0.0219) after 14 days of immersion in these solutions. In the control group, only Transbond™ XT samples showed satisfying color stability (ΔE* < 3.7). Means and standard deviations of L* value, a* value and b* value of L*, a*, b* (LAB) color scale for adhesive samples, according to the solution over time are presented in Supplementary Tables S4–S6.

## 4. Discussion

Coffee and Coca-Cola are very well-known beverages consumed daily around the world. In addition, they have the potential to cause staining of dental materials [9,16]. For this reason, they are among the most frequently used solutions in bracket and adhesive staining studies [17]. Therefore, these solutions, including Cedevita^®^, a familiar vitamin drink in Croatia, were used in this study. The total immersion period was 14 days, since this period is long enough to cause perceptible color modification of ceramic and composite materials, and after this period, a trend towards saturation is expected [5,18]. According to Guler [19], the average time for consumption of 1 cup of coffee is 15 min, and the average consumption is 3.2 cups of coffee per day. Thus, 24 h simulate consumption of the drink over 1 month. Therefore, 14 days of immersion correspond to a period of over one year, which correlates to the duration of orthodontic treatment.

The CIE L* a* b* color system is a recommended method for color change evaluation in the dental field, and it characterizes color based on human perception [20,21]. Most studies accept that differences in color alteration values provided by a spectrophotometer (ΔE*) of 3.7 or more units are considered to be visually perceptible or clinically unacceptable [6,11,14,22,23]. Hence, in this study, all color change values below 3.7 were considered to be satisfactory.

All brackets and adhesive samples changed their color, even in the control group. The fact that color stability was compromised in the colorless Glandosane solution can be explained by water absorption which leads to a decrease in brightness (L* value) [5]. Additionally, Lee et al. [22,24] showed that the color of both the plastic and ceramic brackets will change when they are exposed to thermal cycling—a procedure used to accelerate the aging process and establish color stability of dental materials. This can be associated with the loss of surface finishing characteristics when materials are exposed to the washing effect of saliva [24]. Even though the color was not completely stable, there was only a slight, clinically acceptable change (ΔE* < 3.7) in all bracket brands in the control group, which is in correlation with previous studies [13,23]. Regarding adhesives, Transbond™ XT samples in the control group showed clinically acceptable color change, which is in accordance with a study by Chami et al. [25].

In the present study, coffee had the greatest impact on color stability in almost every bracket brand. This is in correlation with previous studies which confirm that coffee has the highest staining potential [5,13,23]. Moreover, adhesive samples showed the same results in our and other studies [18,26]. According to the literature, coffee might produce more remarkable color changes of the material in comparison to other solutions (e.g., Coca-Cola) even though these solutions have similar color parameters [17]. The reason lies in the fact that coffee solution has a large amount of yellow pigment which is both adsorbed and absorbed in the structure of dental materials and leads to the increase in b* value [5,27]. This penetration and adsorption is probably a result of the compatibility of the polymer phase of the material with yellow pigments in the coffee [14,27].

In this study, all bracket brands and most of the adhesive samples kept in Coca-Cola showed less intense staining. This is in concordance with previous studies [23,28,29]. Moreover, Vapor™ and Chic. brackets showed satisfying color stability after 14 days of immersion (ΔE* < 3.7) in this solution. Although some studies report that lower pH leads to the greatest discoloration due to higher penetration of pigment through altered bracket surface [13,30], others claim that the amount and type of pigment in the solution is the main factor responsible for color alteration [5,31]. Precisely, Coca-Cola cannot promote visible color changes due to the lack of yellow pigment in its composition [14,27].

Contrary to Coca-Cola, Cedevita^®^ is a vitamin drink containing betanin and beta carotene pigments, which are responsible for the yellowish orange color of the drink [32]. It also contains citric acid, which is a weaker acid compared to phosphoric acid in Coca-Cola [33]. Previously mentioned factors can be a possible explanation for the lower bracket discoloration kept in Coca-Cola than in the vitamin drink (Cedevita^®^). However, Transbond™ XT adhesive samples showed the greatest color stability after immersion in this vitamin drink. Thus, more studies are needed to obtain the staining potential of the vitamin drink (Cedevita^®^).

Ceramic brackets can be made of monocrystalline or polycrystalline aluminum oxide. Monocrystalline brackets are translucent, while polycrystalline brackets are non-translucent due to impurities integrated into the manufacturing process and boundaries between the crystals which hinder the passage of light [23,34,35]. Although it was rational to consider that monocrystalline brackets would show higher esthetic performance due to translucency, previous papers obtained different results. Many studies concluded that the same crystalline structure does not follow the same or similar pattern in color alteration when exposed to the same solution under the same conditions and that color stability is highly dependent on the manufacturer brand [13,22,23,24,35]. Our study confirms that since brackets with highest (20/40™ Brackets) and lowest (QuicKlear^®^ III) color stability were both polycrystalline.

The staining potential of the adhesives depends on their composition, surface properties [14,21] and hydrophilicity [36]. The more hydrophilic the resin, the greater the absorption of water and water-soluble pigments [18,25,36]. The color stability of esthetic brackets depends not only on the manufacturing process as mentioned earlier, but also on the surface properties and morphology—size, shape, and thickness of the brackets [11,13,22,35].

Due to the lack of standardization in this kind of study, results cannot be completely comparable, since different solutions, types of brackets and adhesives were used, and various immersion times were specified. It is important to mention that this in vitro study cannot completely imitate the oral cavity conditions. Some important factors which could have an influence on bracket discoloration, such as saliva, oral hygiene maintenance, and biofilm formation, were not included due to the methodological limitations of this study. Therefore, future clinical studies are needed to obtain more accurate results.

## 5. Conclusions

The results of this study indicate that esthetic orthodontic materials are not resistant to discoloration. Color modification occurs in all solutions, even in control groups, and, without doubt, coffee has the greatest impact on color stability. Patients need to be warned that the ingestion of certain drinks can affect the esthetic appearance of their orthodontic brackets.

## Figures and Tables

**Table 1:** Composition, brand and manufacturer of investigated brackets.

Composition	Brand	Manufacturer
Polycrystalline	QuicKlear^®^ III	Forestadent^®^ (Pforzheim, Germany)
Polycrystalline	Vapor™	G&H^®^ Orthodontics (Franklin, IN, USA)
Polycrystalline	Chic.	GC Orthodontics (Breckerfeld, Germany)
Monocrystalline	ClearViz + Mini	DynaFlex^®^ (Lake St. Louis, MO, USA)
Polycrystalline	20/40™ Brackets	American orthodontics (Sheboygan, WI, USA)

**Table 2:** Means and standard deviations of color change values (ΔE*) of ceramic brackets, according to the solution over time.

		Coca-Cola	Coffee	Vitamin Drink	Control
QuicKlear^®^ III	T0-T1	6.60 ± 4.94	7.68 ± 2.06	2.36 ± 0.59	
	T1-T2	8.51 ± 3.66	9.4 ± 1.81	1.13 ± 0.25	
	T2-T3	1.45 ± 1.16	4.89 ± 3.02	6.9 ± 2.23	
	T3-T4	1.31 ± 1.07	2.78 ± 0.95	11.21 ± 2.56	
	T0-T4	17.14 ± 2.68 **ª**	23.26 ± 1.29 **ª**	17.51 ± 2.43 **ª**	2.45 ± 1.16
ClearViz + Mini	T0-T1	2.34 ± 1.81	3.92 ± 0.39	4.85 ± 1.25	
	T1-T2	1.08 ± 0.41	11.5 ± 1.17	3.68 ± 0.95	
	T2-T3	1.39 ± 0.66	6.44 ± 2.02	5.65 ± 0.76	
	T3-T4	2.7 ± 1.31	1.68 ± 1.82	2.63 ± 1.77	
	T0-T4	4.36 ± 3.16	20.03 ± 5.09	9.64 ± 2.46 **ª**	1.88 ± 0.62
Chic.	T0-T1	1.35 ± 0.80	9.36 ± 0.81	7.41 ± 1.05	
	T1-T2	1.19 ± 0.3	3.8 ± 0.21	2.09 ± 1.25	
	T2-T3	1.09 ± 0.46	8.1 ± 1.0	2.12 ± 1.6	
	T3-T4	1.01 ± 0.77	6.95 ± 2.39	4.18 ± 1.35	
	T0-T4	2.31 ± 0.47 **ª**	22.77 ± 2.69 **ª**	11.88 ± 0.76	1.51 ± 0.74
Vapor™	T0-T1	1.83 ± 0.6	2.13 ± 0.15	5.03 ± 0.84	
	T1-T2	1.35 ± 0.43	8.81 ± 2.07	1.56 ± 0.59	
	T2-T3	0.77 ± 0.48	8.9 ± 1.7	1.51 ± 0.48	
	T3-T4	0.78 ± 0.2	2.09 ± 0.84	0.9 ± 0.6	
	T0-T4	3.38 ± 0.41	19.08 ± 0.59	7.79 ± 0.18 **ª**	2.14 ± 0.5
20/40™ Brackets	T0-T1	3.65 ± 0.33	4.24 ± 0.24	3.52 ± 0.45	
	T1-T2	4.97 ± 0.84	4.65 ± 0.27	6.52 ± 0.86	
	T2-T3	1.09 ± 0.7	2.17 ± 0.64	2.66 ± 0.82	
	T3-T4	0.71 ± 0.41	3.78 ± 0.69	2.2 ± 0.62	
	T0-T4	5.9 ± 0.09	11.74 ± 0.25 **ª**	12.5 ± 1.0	2.04 ± 0.56

**ª** Statistically significant.

**Table 3:** Means and standard deviations of color change values (ΔE*) of adhesive samples, according to the solution over time.

ΔE*		Coca-Cola	Coffee	Vitamin Drink	Control
Transbond™ XT	T0-T1	4.72 ± 1.65	10.31 ± 1.53	7.7 ± 1.82	
	T1-T2	2.58 ± 1.13	3.21 ± 1.58	3.52 ± 1.49	
	T2-T3	3.02 ± 0.71	13.27 ± 1.19	2.9 ± 0.84	
	T3-T4	8.43 ± 1.39	6.04 ± 1.25	1.38 ± 0.52	
	T0-T4	13.58 ± 1.07 **ª**	27.6 ± 0.58	11.47 ± 0.51 **ª**	2.43 ± 0.16
BracePaste^®^ color change adhesive	T0-T1	11.55 ± 0.28	15.64 ± 2.32	12.61 ± 0.34	
	T1-T2	4.97 ± 1.0	1.32 ± 0.24	1.6 ± 0.17	
	T2-T3	328 ± 2.36	6.76 ± 2.18	9.34 ± 0.53	
	T3-T4	3.34 ± 0.34	2.35 ± 1.21	2.72 ± 0.36	
	T0-T4	20.14 ± 0.23 **ª**	22.81 ± 2.57	20.79 ± 0.61 **ª**	7.77 ± 1.22
BracePaste^®^ adhesive	T0-T1	3.94 ± 0.9	14.7 ± 1.31	4.59 ± 0.17	
	T1-T2	4.02 ± 0.69	3.41 ± 0.74	1.9 ± 0.4	
	T2-T3	2.27 ± 0.42	2.21 ± 0.83	11.35 ± 0.79	
	T3-T4	9.62 ± 0.66	5.04 ± 0.27	2.39 ± 0.58	
	T0-T4	15.9 ± 0.63	20.93 ± 0.69	19.11 ± 0.27	4.11 ± 2.8

**ª** Statistically significant.

## Data Availability

Not applicable.

## Supplementary Materials

The following supporting information can be downloaded at: https://www.mdpi.com/2304-6767/10/7/115/s1, Table S1: Means and standard deviations of L* value of L*, a*, b* (LAB) color scale for ceramic brackets, according to the solution over time, Table S2: Means and standard deviations of a* value of L*, a*, b* (LAB) color scale for ceramic brackets, according to the solution over time, Table S3: Means and standard deviations of b* value of L*, a*, b* (LAB) color scale for ceramic brackets, according to the solution over time, Table S4: Means and standard deviations of L* value of L*, a*, b* (LAB) color scale for adhesive samples, according to the solution over time, Table S5: Means and standard de-viations of a* value of L*, a*, b* (LAB) color scale for adhesive samples, according to the solution over time, Table S6: Means and standard deviations of b* value of L*, a*, b* (LAB) color scale for adhesive samples, according to the solution over time.
