# Supplementary material for: Color Stability of Orthodontic Ceramic Brackets and Adhesives in Potentially Staining Beverages—In Vitro Study
Source: Dent J (Basel). 2022 Jun 22;10(7):115. doi: 10.3390/dj10070115 (PMC9351677; doi:10.3390/dj10070115)
Supplement: Supplementary file 1 [file dentistry-10-00115-s001.zip › dentistry-1777878-supplementary.pdf]

Table S1. Means and standard deviations of L* value of L*, a*, b* (LAB) color scale for ceramic brackets, according to the solution over time

L parameter		**Coca Cola**	**Coffee**	**Vitamin drink**	**Control**
QuicKlear® III	T0	65.73 ± 3.68 ^{ab}	58.56 ± 1.12 ^{ab}	66.1 ± 2.62 ^{ab}	67.26 ± 1.31 ^{ab}
	T1	59.35 ± 3.33 ^{ab}	51.95 ± 1.23 ^{ab}	66.55 ± 2.95 ^{ab}	65.35 ± 0.81 ^{ab}
	T2	51.15 ± 1.07 ^{ab}	42.71 ± 1.24 ^{ab}	66.78 ± 3.25 ^{ab}	65.16 ± 1.13 ^{ab}
	T3	49.86 ± 0.17 ^{ab}	38.5 ± 2.53 ^{ab}	59.95 ± 3.09 ^{ab}	71.74 ± 4.2 ^{ab}
	T4	48.93 ± 1.35 ^{ab}	36.38 ± 0.62 ^{ab}	48.76 ± 0.86 ^{ab}	69.15±2.08 ^{ab}
ClearViz+Mini	T0	96.27 ± 2.85 ^{ac}	96.13 ± 2.95 ^{ab}	96.66 ± 2.3 ^{a}	96.6 ± 2.13 ^{ac}
	T1	95.21 ± 0.36 ^{ac}	94.44 ± 1.45 ^{ac}	99.34 ± 0.97 ^{ac}	97.39 ± 0.87 ^{ac}
	T2	94.15 ± 0.19 ^{ac}	83.26 ± 0.93 ^{a}	95.72 ± 0.24 ^{ab}	97.39 ± 1.52 ^{a}
	T3	94.32 ± 1.57 ^{ac}	78.87 ± 1.03 ^{a}	93.47 ± 1.53 ^{ac}	96.47 ± 1.6 ^{a}
	T4	92.09 ± 0.49 ^{ac}	78.17 ± 2.94 ^{a}	92.41 ± 1.59 ^{ac}	96.93 ± 0.79 ^{ac}
Chic.	T0	83.65 ± 0.64 ^{c}	83.66 ± 3.33 ^{b}	83.79 ± 0.98	86.44 ± 1.66
	T1	84.17 ± 0.54 ^{c}	83.27 ± 0.86 ^{c}	83.23 ± 0.70 ^{c}	86.79 ± 0.49
	T2	84.4 ± 0.49	79.51 ± 0.77	82.21 ± 0.58 ^{b}	84.29 ± 0.29
	T3	84.53 ± 0.24	74.39 ± 2.03	80.2 ± 1.1 ^{c}	83.23 ± 0.13
	T4	85.41 ± 0.63	67.75 ± 0.71	76.06 ± 0.51^{c}	85.48 ± 0.76
Vapor™	T0	91.67 ± 0.74 ^{b}	90.25 ± 0.26 ^{b}	92.1 ± 0.14 ^{bc}	93.44 ± 0.78 ^{b}
	T1	90.83 ± 0.12 ^{b}	90.13 ± 0.42 ^{b}	91.27 ± 0.31 ^{bc}	91.83 ± 0.92 ^{b}
	T2	90.11 ± 0.32 ^{b}	81.43 ± 2.02 ^{b}	90.52 ± 0.82 ^{b}	92.13 ± 2.2 ^{b}
	T3	90.31 ± 0.2 ^{b}	75.85 ± 1.57	90.09 ± 0.46 ^{bc}	96.68 ± 0.73 ^{b}
	T4	89.8 ± 0.15 ^{b}	74.38 ± 0.49	89.55 ± 0.57 ^{b}	93.71 ± 1.2 ^{b}
20/40™ Brackets	T0	85.07 ± 0.41	84.38 ± 0.33	85.28 ± 0.42	84.6 ± 0.37 ^{c}
	T1	84.81 ± 0.41	83.79 ± 0.40	85.05 ± 0.5	84.64 ± 0.76
	T2	83.59 ± 0.73 ^{c}	79.29 ± 0.26	84.47 ± 0.29	84.34 ± 0.42
	T3	83.06 ± 0.43 ^{c}	78.69 ± 0.36 ^{b}	83,12 ± 0,69	83.59 ± 0.58
	T4	82.64 ± 0.35 ^{c}	77.32 ± 0.22 ^{b}	81,57 ± 0,40	83.38 ± 0.74 ^{c}

Table S2. Means and standard deviations of a* value of L*, a*, b* (LAB) color scale for ceramic brackets, according to the solution over time

a parameter		Coca Cola	Coffee	Vitamin drink	Control
	T0	0.24 ± 0.07 [a]	0.59 ± 0.07	0.17 ± 0.38 [a]	-0.24 ± 0.37 [a b]
	T1	-0.2 ± 0.25 [a]	1.08 ± 0.24 [a]	0.57 ± 0.26 [a]	0.89 ± 0.89
QuicKlear® III	T2	-0.05 ± 0.54	1.45 ± 0.31	0.87 ± 0.15	0.4 ± 0.1
	T3	0.2 ± 0.11	2.51 ± 0.39	1.05 ± 0.34	0.01 ± 0.31 [a b]
	T4	-0.11 ± 0.04 [a c]	2.34 ± 0.37 [a]	0.57 ± 0.2	0.22 ± 0.18 [a b]
	T0	0.13 ± 0.2 [b c]	0.47 ± 0.23	0.25 ± 0.05 [b]	0.23 ± 0.1 [c]
	T1	0.23 ± 0.05 [b]	0.92 ± 0.08 [b]	0.75 ± 0.23 [b]	0.68 ± 0.12
ClearViz+Mini	T2	0.36 ± 0.04	1.17 ± 0.39 [a]	0.37 ± 0.04 [a]	0.37 ± 0.09
	T3	0.71 ± 0.06	4.22 ± 0.26 [a]	0.91 ± 0.1	0.22 ± 0.02 [c]
	T4	0.19 ± 0.01	4.29 ± 0.31	1.45 ± 0.81	0.28 ± 0.06 [c d]
	T0	1.18 ± 0.19 [a b]	2.27 ± 0.55 [a]	1.52 ± 0.45 [a b]	1.25 ± 0.21 [a]
	T1	0.97 ± 0.51	2.67 ± 0.22	2.2 ± 0.43 [a b]	0.46 ± 0.03 [a]
Chic.	T2	1.22 ± 0.72 [a]	2.34 ± 0.2 [b]	2.11 ± 0.1 [a b]	-0.09 ± 0.23 [a]
	T3	0.34 ± 0.66	4.52 ± 0.72 [b]	1.95 ± 0.11 [a b]	0.83 ± 0.05 [a]
	T4	0.65 ± 0.27 [b]	4.52 ± 0.36	2.23 ± 0.06 [a b]	1.07 ± 0.11 [a c]
	T0	1.01 ± 0.22 [c]	0.71 ± 0.04 [b]	0.69 ± 0.11	1.73 ± 0.31 [b c]
	T1	1.24 ± 0.05	1.03 ± 0.07 [c]	0.7 ± 0.07	1.19 ± 0.17 [a]
Vapor™	T2	0.83 ± 0.42 [b]	0.63 ± 0.05 [b c]	0.61 ± 0.1	1.16 ± 0.2 [a]
	T3	1.19 ± 0.47 [a]	1.09 ± 0.12 [a b]	0.2 ± 0.0 [a]	1.21 ± 0.08 [b c]
	T4	1.33 ± 0.04 [a c]	1.86 ± 0.12 [b]	0.45 ± 0.1 [a]	1.29 ± 0.29 [b d]
	T0	0.6 ± 0.07 [a]	0.16 ± 0.22 [a b]	-0.3 ± 1.5	0.56 ± 0.04
	T1	2.12 ± 0.22 [b]	3.7 ± 0.10 [a b c]	0.6 ± 0.64	0.6 ± 0.0
20/40™ Brackets	T2	-2.63 ± 0.58 [a b]	3.59 ± 0.44 [a c]	0.08 ± 0.44 [b]	0.45 ± 0.04
	T3	-3.24 ± 0.44 [a]	3.31 ± 0.33	-0.94 ± 0.59 [b]	0.5 ± 0.1
	T4	-3.6 ± 0.45 [b c]	6.32 ± 0.48 [a b]	0.24 ± 0.36 [b]	0.5 ± 0.1

Table S3. Means and standard deviations of b* value of L*, a*, b* (LAB) color scale for ceramic brackets, according to the solution over time

b parameter		Coca Cola	Coffee	Vitamin drink	Control
QuicKlear® III	T0	2.47 ± 0.07	3.13 ± 0,17 ^{ab}	3.6 ± 0.20 ^{ab}	2.09 ± 0.5
	T1	3.55 ± 0.2	6.75 ± 1,89	5.75 ± 0.69 ^{a}	2.7 ± 0.36
	T2	5.47 ± 0.46 ^{a}	8.0 ± 1,12 ^{a}	6.24 ± 0.39 ^{a}	2.78 ± 0.07
	T3	5.81 ± 0.05 ^{ab}	9.85 ± 0,64	5.55 ± 0.52 ^{ab}	2.97 ± 0.25
	T4	5.75 ± 0.67 ^{ab}	9.91 ± 0,12	5.93 ± 0.1 ^{ab}	2.93 ± 0.28
ClearViz+Mini	T0	2.23 ± 0.34	1.5 ± 0,39 ^{a}	1.96 ± 0.09 ^{a}	2.19 ± 0.29
	T1	2.11 ± 0.2 ^{a}	4.43 ± 1,02	5.78 ± 0.45	5.1 ± 0.43 ^{a}
	T2	2.21 ± 0.24 ^{ab}	6.81 ± 1,82	5.59 ± 0.07 ^{b}	3.13 ± 0.29
	T3	2.04 ± 0.29 ^{ac}	9.77 ± 1,18	10.62 ± 0.32	1.43 ± 0.1 ^{abc}
	T4	2.99 ± 0.56 ^{ac}	9.33 ± 0,66 ^{a}	10.13 ± 1.11	2.29 ± 0.18 ^{ab}
Chic.	T0	2.87 ± 0.31 ^{a}	2.4 ± 0,17	2.45 ± 0.46	2.97 ± 0.26 ^{ab}
	T1	3.64 ± 0.68	11.33 ± 0,36 ^{a}	9.55 ± 0.81 ^{ab}	3.68 ± 0.08 ^{b}
	T2	4.13 ± 0.42	11.31 ± 0,33 ^{bc}	11.16 ± 1.21 ^{bc}	2.69 ± 0.11
	T3	3.97 ± 0.47 ^{b}	16.88 ± 2,0 ^{a}	11.13 ± 0.77 ^{a}	3.37 ± 0.16 ^{a}
	T4	4.23 ± 0.15 ^{b}	18.43 ± 0,96 ^{ab}	11.36 ± 0.45 ^{a}	3.08 ± 0.13
Vapor™	T0	1.71 ± 0.23 ^{a}	1.31 ± 0,01 ^{b}	1.42 ± 0.1 ^{b}	1.89 ± 0.13 ^{a}
	T1	3.19 ± 0.3 ^{b}	3.33 ± 0,09 ^{a}	6.35 ± 0.95	2.03 ± 0.49 ^{a}
	T2	4.12 ± 0.16	4.61 ± 0,1 ^{ab}	7.48 ± 0.36	3.31 ± 0.44 ^{b}
	T3	4.13 ± 0.04	10.99 ± 0,13 ^{b}	8.39 ± 0.14 ^{c}	3.34 ± 0.2 ^{b}
	T4	4.43 ± 0.12	11.83 ± 0,5	8.76 ± 0.11 ^{c}	3.28 ± 0.39 ^{a}
20/40™ Brackets	T0	2.45 ± 0.14	2.26 ± 0,27	2.21 ± 0.33	1.68 ± 0.25 ^{b}
	T1	5.7 ± 0.3 ^{ab}	4.40 ± 0,10	5.34 ± 0.14 ^{b}	3.12 ± 0.09
	T2	5.45 ± 0.18 ^{b}	5.5 ± 0,1 ^{c}	11.77 ± 0.94 ^{ac}	3.2 ± 0.19
	T3	5.4 ± 0.1 ^{c}	7.52 ± 0,57 ^{ab}	13.61 ± 1.62 ^{bc}	3.41 ± 0.21 ^{c}
	T4	5.71 ± 0.11 ^{c}	9.32 ± 0,08 ^{b}	14.04 ± 1.55 ^{bc}	3.25 ± 0.2 ^{b}

Table S4. Means and standard deviations of L* value of L*, a*, b* (LAB) color scale for adhesive samples, according to the solution over time

L parameter		Coca Cola	Coffee	Vitamin drink	Control
Transbond™ XT	T0	74.35 ± 0.55 [a]	73.87 ± 0.16	74.57 ± 0.26 [a]	67.55 ± 0.11 [a]
	T1	71.97 ± 0.94 [a]	67.48 ± 0.29 [a]	71.05 ± 0.66 [a]	70.55 ± 0.65 [a]
	T2	69.91 ± 0.25 [a]	64.74 ± 1.55	69.21 ± 0.81 [a]	71.45 ± 0.74 [a]
	T3	68.36 ± 0.46 [a]	55.99 ± 1.19 [a]	70.26 ± 0.36 [a]	70.96 ± 0.39 [a]
	T4	60.63 ± 0.95 [a]	50.67 ± 0.61 [a]	70.63 ± 0.53 [a]	72.01 ± 0.09 [a]
BracePaste® adhesive color change	T0	75.41 ± 0.1	74.26 ± 1.16 [a]	75.67 ± 0.03	74.97 ± 0.78
	T1	74.47 ± 0.44	68.1 ± 0.47	75.21 ± 0.18	75.06 ± 0.31
	T2	70.11 ± 0.27	69.01 ± 0.2	73.99 ± 0.07	75.54 ± 0.29
	T3	69.63 ± 0.43	64.5 ± 0.78	74.36 ± 0.22	74.8 ± 1.16
	T4	69.47 ± 0.23	65.14 ± 0.4	71.82 ± 0.4	76.89 ± 0.23
BracePaste® adhesive	T0	81.94 ± 0.07 [a]	81.16 ± 0.5 [a]	81.89 ± 0.11 [a]	81.7 ± 0.41 [a]
	T1	80.16 ± 0.16 [a]	73.15 ± 0.37 [a]	80.98 ± 0.24 [a]	89.52 ± 0.75 [a]
	T2	77.22 ± 0.11 [a]	74.82 ± 1.1 [a]	80.14 ± 0.05 [a]	80.23 ± 0.22 [a]
	T3	76.66 ± 0.13 [a]	72.97 ± 0.07 [a]	76.13 ± 1.25 [a]	79.58 ± 1.45 [a]
	T4	72.88 ± 0.28 [a]	68.74 ± 0.23 [a]	76.49 ± 0.42 [a]	79.96 ± 1.37 [a]

Table S5. Means and standard deviations of a* value of L*, a*, b* (LAB) color scale for adhesive samples, according to the solution over time

a parameter		Coca Cola	Coffee	Vitamin drink	Control
Transbond™ XT	T0	0.78 ± 0.13 [a]	0.76 ± 0.12	0.91 ± 0.04 [a]	0.82± 0.05 [a]
	T1	0.89 ± 0.71	1.43 ± 0.21	2.14 ± 0.74 [a]	-0.06 ± 0.25
	T2	0.63 ± 0.08	1.24 ± 0.36	1.93 ± 0.09 [a]	1.25 ± 1.28 [a]
	T3	0.76 ± 0.22	3.89 ± 0.18 [a]	1.51 ± 0.59 [a b]	0.84 ± 0.14 [a]
	T4	1.29 ± 0.21	3.91 ± 0.14 [a]	1.57 ± 0.42	0.89 ± 0.02 [a]
BracePaste® adhesive color change	T0	0.43 ± 0.07	1.08 ± 0.44 [a]	0.46 ± 0.05	0.13 ± 0.06
	T1	1.67 ± 0.03 [a]	2.78 ± 0.1 [a]	1.89 ± 0.05	0.7 ± 0.09 [a]
	T2	1.67 ± 0.06 [a]	2.41 ± 0.07 [a]	1.79 ± 0.02	0.4 ± 0.09
	T3	2.24 ± 0.08 [a]	3.53 ± 0.52	1.44 ± 0.14 [b]	0.02 ± 0.12
	T4	2.24 ± 0.05 [a]	3.62 ± 0.2	1.86 ± 0.04 [a]	-0.06 ± 0.11
BracePaste® adhesive	T0	-3.79 ± 1.9	-4.59 ± 0.2 [a]	-4.8 ± 0 [a]	-4.99 ± 0.11 [a]
	T1	-4.38 ± 0.02 [a]	-2.9 ± 0.3 [a]	-2.76 ± 0.28 [a]	-4.48 ± 0.11 [a]
	T2	-3.71 ± 0.07 [a]	-1.57 ± 0.23 [a]	-2.84 ± 0.26 [a]	-2.97 ± 0.15 [a]
	T3	-3.1 ± 0.1 [a]	-2.08 ± 0.04 [a]	-1.31 ± 0.31 [a]	-3.67 ± 0.66 [a]
	T4	-2.18 ± 0.11 [a]	-1.19 ± 0.08 [a]	-0.86 ± 0.02 [a]	-3.06 ± 1.58 [a]

Table S6. Means and standard deviations of b* value of L*, a*, b* (LAB) color scale for adhesive samples, according to the solution over time

b parameter		**Coca Cola**	**Coffee**	**Vitamin drink**	**Control**
Transbond™ XT	T0	0.82 ± 0.55 [a]	12.03 ± 0.05 [a]	0.78 ± 0.13 [a]	0.91± 0.04 [a b]
	T1	8.34 ± 1.56 [a]	12.03 ± 0.05 [a]	10.43 ± 2.29	4.68 ± 0.98
	T2	7.34 ± 0.38 [a]	11.63 ± 0.89 [a]	12.79 ± 0.71	6.2 ± 0.21 [a]
	T3	4.83 ± 0.94 [a]	21.29 ± 0.65	15.17 ± 0.51 [a]	5.97 ± 0.63 [a]
	T4	2.89 ± 0.38 [a]	18.56 ± 0.47 [a]	14.59 ± 0.62 [a]	4.8 ± 0.08 [a]
BracePaste® adhesive color change	T0	7.56 ± 0.22 [a]	9.99 ± 1.76 [a]	7.7 ± 0.09 [a]	6.04 ± 0.5 [a]
	T1	18.99 ± 0.44 [a]	24.27 ± 0.56 [a]	20.22 ± 0.42 [a]	6.27 ± 0.47
	T2	20.23 ± 2.44 [a]	28.83 ± 0.43 [a]	19.36 ± 0.25 [a]	14.67 ± 0.5 [a]
	T3	23.39 ± 0.32 [a]	28.67 ± 1.73 [a]	28.67 ± 0.3 [a]	14.63 ± 0.59 [a]
	T4	26.71 ± 0.1[a]	30.74 ± 0.49 [a]	28.07 ± 0.49 [a]	13.56 ± 0.53 [a]
BracePaste® adhesive	T0	5.69 ± 0.07	5.23 ± 0.85	5.88 ± 0.22	6.09 ± 0.5 [b]
	T1	8.83 ± 0.67	17.41 ± 0.85	9.87 ± 0.53 [a]	7.04 ± 0.33 [a]
	T2	11.38 ± 0.52	19.42 ± 0.93	11.47 ± 1.08 [a]	11.6 ± 0.6
	T3	9.49 ± 0.18	19.13 ± 0.03 [a]	21.89 ± 0.44	8.2 ± 2.49
	T4	18.61 ± 0.44	21.72 ± 0.37	23.78 ± 0.25	9.13 ± 2.24
